# Antibiotics, Multidrug-Resistant Bacteria, and Antibiotic Resistance Genes: Indicators of Contamination in Mangroves?

**DOI:** 10.3390/antibiotics10091103

**Published:** 2021-09-13

**Authors:** Oskar A. Palacios, Jaime Raúl Adame-Gallegos, Blanca Estela Rivera-Chavira, Guadalupe Virginia Nevarez-Moorillon

**Affiliations:** Facultad de Ciencias Químicas, Universidad Autónoma de Chihuahua, Circuito Universitario s/n Campus Universitario II, Chihuahua 31125, Mexico; opalacios@uach.mx (O.A.P.); jadame@uach.mx (J.R.A.-G.); bchavira@uach.mx (B.E.R.-C.)

**Keywords:** mangroves, antibiotics, multidrug-resistant bacteria, aquaculture, tourism

## Abstract

Multidrug-resistant bacteria and antibiotic resistance genes can be monitored as indicators of contamination in several environments. Mangroves are among the most productive ecosystems, and although they can be resilient to the action of climate phenomena, their equilibrium can be affected by anthropogenic activities. Regarding the presence and persistence of multidrug-resistant bacteria in mangroves, it is common to think that this ecosystem can function as a reservoir, which can disperse the antibiotic resistance capacity to human pathogens, or serve as a filter to eliminate drug-resistant genes. The possible impact of anthropogenic activities carried out near mangroves is reviewed, including wastewater treatment, food production systems, leisure, and tourism. Adverse effects of antibiotic resistance genes or multidrug-resistant bacteria, considered as emerging contaminants, have not been reported yet in mangroves. On the contrary, mangrove ecosystems can be a natural way to eliminate antibiotics, antibiotic-resistant bacteria, and even antibiotic-resistant genes from the environment. Although mangroves’ role in decreasing antibiotics and antibiotic resistance genes from the environment is being proposed, the mechanisms by which these plants reduce these emerging contaminants have not been elucidated and need further studies. Additionally, further evaluation is needed on the effects of antibiotics and antibiotic-resistant bacteria in mangroves to generate an analysis of the human contribution to the degradation of this specific ecosystem as well as to define if these contaminants can be used as indicators of contamination in mangrove ecosystems.

## 1. Introduction

Mangroves are coastal ecosystems that are located in the interphase of marine and terrestrial environments, particularly in the tropical and subtropical regions, in humid and even arid zones. There are 152,000 km^2^ of mangroves distributed in 123 countries worldwide [1]. Over 62 plant species including trees, shrubs, palms, and mangroves are found in the mangrove ecosystem; all the plants have common traits that allow them to survive periodic flooding, high salinity, and anoxic sediments [1]. These ecosystems offer many environmental advantages to human populations, such as carbon sequestration [2,3], nutrient sinks facilitated by denitrification and nitrogen fixation [4], influence on the structure and conservation of marine communities [5,6], and aiding in the stabilization of coastlines due to wave attenuation [7]. Mangroves are also a source of wood [8] and an important travel and tourism destination [9]. Due to all these environmental services, there is a considerable economic value associated with mangrove ecosystems. Carbon sequestration alone was valued at USD 13,000–23,000 per hectare in mangroves of South Florida [10], while in Baja California, where the mangroves are short and developed in arid and nutrient-poor landscapes, a Blue Carbon project was valued at USD 426,000 per year [4]. Regardless of the benefits and economic value of mangroves to their close communities, the ecosystem is not considered an attractive environment, mainly due to the perception of mangroves as hostile, foul-smelling, and muddy [11]. Because of the requirements of a growing human population, mangrove equilibrium is under pressure by human activities, such as land conversion, overharvesting, and pollution [6,12]. As a result, one-quarter of the world’s mangroves have been lost due to human action [7].

The main indicators used for conservation or perturbation in mangrove ecosystems are based on image analysis of the several plants present. The most recognized variable that indicates the condition of mangroves is the vegetation structure, which involves leaf area index, canopy height, and standing biomass [13]. The fluctuating asymmetry (random differences in size and shape between the two sides of a bilateral character) has also been recognized as an indicator of the perturbation/conservation of mangrove trees [14]. On the other hand, ants and phytoplankton diversity, crabs, and periwinkle, the latter for heavy metal pollution, have been proposed as conservation or perturbation indicators of mangroves [15,16,17]. Moreover, for the mangrove process, nitrogen fixation has been proposed not only for conservation/perturbation conditions but also as a restoration indicator [18].

Emerging contaminants (ECs) such as antibiotic residues and antibiotic-resistant bacteria (ARB) are critical problems that have been reported lately in many different environments. Although these contaminants are not new, their presence in several environments has increased along with the increment in industrialized areas and crowded communities [19]. Their presence in the environment has been related to human activities such as industrial and domestic wastewater, human and veterinary medicine, and agriculture [20]. Because mangroves are ecosystems that can be directly or indirectly affected by human activities in coastlines, there is an interest in the identification of the possible effects of the anthropogenic activities near these ecosystems, in the presence of emerging contaminants.

Today, the effect of anthropogenic activities in mangroves is contradictory. While the presence of ARB and antibiotic resistance genes (ARGs) in mangroves from India was related to anthropogenic activities [21], in other studies, mangroves have been considered as “natural wastewater wetlands” with an active role as nutrient filters and even to reduce the presence of ARG and antibiotics (ABs) in these ecosystems [22,23,24]. Therefore, several questions have arisen: (1) What are the uptake mechanisms developed by mangrove plants to reduce antibiotics and their degradation products from sediments?; (2) How do antibiotic compounds affect the physiology of mangroves?; (3) What are the biological mechanisms developed by mangroves to mitigate the stress produced by these types of ECs?; and finally, with more data about the real effect of antibiotics, ARB, and ARGs in mangrove ecosystems, (4) Could these ECs be considered as contaminant indicators in these ecosystems? Regardless of the lack of information, as these emerging contaminants are present in the environment, they can be a source of contamination for food and water and will eventually be in contact again with the human population, thus closing the cycle [25]. Hence, this review aims to analyze the possible paths of emerging contaminants related to different human activities, specifically antibiotic residues, ARB, and ARGs in mangroves, as well as, based on other similar models (wetlands), propose the possible mechanisms of mangroves to reduce the presence of ECs in the ecosystem.

## 2. Results

**Figure 1:**
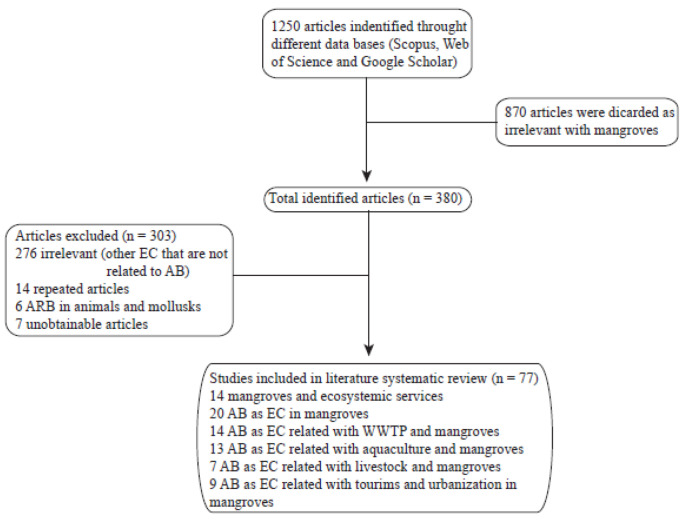
Flow diagram of identification and selection process included in systematic review.

A total of 1250 papers were obtained from the three different databases, and 870 were discarded as irrelevant to mangroves as they focused on wetlands or other water bodies. From the remaining 380 papers, 276 were discarded since they were dealing with other emerging contaminants such as heavy metals, hormones, or isolate microorganisms with antibiotic production. From the last 104 papers, 14 were repeated articles, 6 focused on the detection of antibiotic-resistant bacteria in animals or mollusks, and 7 were unavailable. The last 77 documents between papers and book chapters were analyzed and used for this review (Figure 1). Since information on ARB and ARGs in mangroves is minimal, the fate of these ECs was also reviewed in wetlands to discuss the possible mechanisms of degradation or disposal of these contaminants in the environment.

## 3. Discussion

### 3.1. Possible Paths of Emerging Contaminants (ABs, ARB, and ARGs) in the Environment

**Figure 2:**
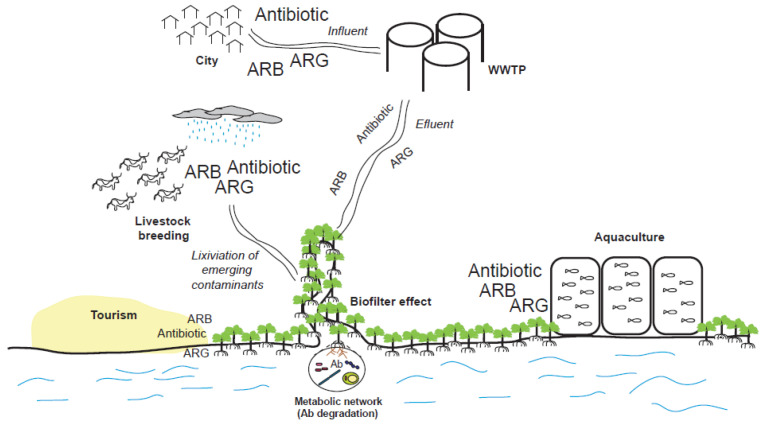
Anthropogenic activities that can influence the presence of emerging contaminants related to antibiotics in mangroves. Legend: Ab: Antibiotic. ARG: Antibiotic resistance gene. ARB: Antibiotic-resistant bacteria. WWTP: Wastewater treatment plant.

The era of antibiotic therapy has led to an increase in the concentration of these compounds in the environment. Even more, the efforts to develop novel antibiotics have also increased the diversity of chemical structures with antibiotic capacities. Because of the dispersal of antibiotics in the environment, there is an increase in ARB as well as ARGs (Figure 2). The combination of biotic and abiotic factors that interact in different environments can have a differential impact on the accumulation or degradation of ECs, but is not exclusive to a particular ecosystem. Therefore, ECs in mangroves can have a similar behavior as in other aquatic ecosystems or even in artificially constructed ecosystems, such as wastewater treatment plants (WWTPs). Table 1 includes recent reports on the presence of ARB or ARGs in the environment, related to human activities, in relation to the mangrove ecosystem.

#### 3.1.1. Urban Wastewater

Wastewater is recognized as one of the most common distribution lines of ECs related to antibiotic resistance (antibiotic residues, ARB, and ARGs) since wastewater treatment plants (WWTPs) have shown high variability in their capacity of antibiotic reduction. Depending on the season, WWTP efficiencies can vary from 66–97% in winter to 17–66% in autumn, while ARB prevalence has increased in effluents (up to 2 logs), reaching a value of 80% of the total bacteria. Similarly, the ARG presence in effluents of WWTPs shows a considerable increment compared with values in influents [26]. The dissemination of antibiotic residues by effluents of wastewater treatment plants is a reality since only an average of 75% of the antibiotics are removed within the process. Even with the use of membrane technologies, ARGs are still detected in values that reach 10^3^ GCN·mL^−1^ effluents [27]. Moreover, ARGs are prevalent during all the wastewater processing lines, and even ARB can survive to different disinfection treatments, reaching release concentrations in effluents of 1015 CFU·d^−1^ and 1018 GCN·d^−1^ [28,29].

On the other hand, mobile genetic elements such as transposons, plasmids, and integrons are abundant in parts of the wastewater treatment process, including activated and sewage sludge [30]. For the latter, wastewater treatment plant effluents are recognized as antibiotic resistance reservoirs due to selective pressure generated in the process [31]. Wetlands with different plant species used in wastewater treatment enable the removal and recovery of valuable nutrients, such as nitrogen and phosphorous, used for plant growth. The implementation of wetlands at the end of aerobic wastewater treatment systems has been proposed to enhance the removal of ECs from effluents, since up to 90% of antibiotic residues and ARB are removed [32]. However, a fluctuation in the antibiotic resistance profile of microorganisms in wastewater has been observed with the use of wetlands as tertiary treatment, which indicates a potential effect of plant–bacteria interaction on the prevalence of antibiotic resistance [33].

Mangroves have been considered as “natural wastewater wetlands” present in coastlines and could act as active nutrient filters from runoff water from cities or human communities. Moreover, mangrove plants have been proposed since the 1990s as part of the secondary treatment system for municipal wastewater in China. The proposal considers the advantage of both oxygenic and anoxic conditions that these plants develop in their roots, allowing oxidation and reduction processes by microorganisms from the mangrove rhizosphere for the removal and transformation of pollutants [22]. Nevertheless, high concentrations of antibiotics present in mangrove ecosystems due to human activities carried out in their proximity can affect microbial activity [23] and facilitate the appearance of multidrug-resistant (MDR) bacteria [34]. The effect of domestic wastewater discharges in the presence of antibiotics, ARGs, or even ARB in mangroves has not been extensively documented. In their study, Ghaderpour and coworkers [34] demonstrated that domestic wastewater discharges were related to the presence of an MDR strain of *E. coli* (with a resistance prevalence to beta-lactams downstream the villages) in a close-by mangrove area. This correlation could be considered proof of the impact of the effluents from wastewater treatment systems on this type of ecosystem. Nonetheless, more information is necessary to evaluate the direct effect of wastewater treatment in the presence of ECs on mangrove ecosystems.

**Table 1:** Recent studies (2015–2020) on the relation between anthropogenic activities and presence of antibiotics, antibiotic resistance genes (ARGs), and antibiotic-resistant bacteria (ARB) in mangroves.

Activity	Source	Antibiotic, ARG, or ARB	Reference
Wastewater treatment	Water and sediment	ARB: *E. coli* tested to gentamicin, kanamycin, streptomycin, neomycin, amoxicillin/clavulanic acid, ampicillin, ceftriaxone, ceftiofur, nalidixic acid, oxolinic acid, enrofloxacin, ciprofloxacin, tetracycline, chloramphenicol and sulfamethoxazole/trimethoprim ARG: Integrase genes class 1, 2, and 3	[34]
	Sediment	ARG: *mtrA, rpoB, rpoC, rpsL, ef-Tu, par-Y*	[24]
Aquaculture	Water and sediment	Antibiotic compounds: sulfonamides, fluoroquinolones, tetracyclines and chloramphenicol	[23]
	Sediment and plant	Antibiotic compounds: norfloxacin, ciprofloxacin and enrofloxacin	[35]
	Water and sediment	Antibiotic compounds: chloramphenicol ARG: *cata1, cata2, cml_e1*, and *cml_e3*	[36]
Livestock breeding	Sediment	Antibiotic compounds: quinolone	[37]
Urbanization	Sediment	*blaTEM* gene; ARB to ampicillin, kanamycin, vancomycin, and tetracycline	[21]
	Sediment	ARB to ampicillin, gentamicin, chloramphenicol, ciprofloxacin, tetracycline, vancomycin, methicillin	[38]

#### 3.1.2. Aquaculture

The increasing demand for food supply due to continuous human population growth requires the establishment of intensive production systems, and one example is the modern aquaculture industry. In intensive aquaculture methods, bacterial fish diseases are common. Consequently, the use of antibiotics has increased, reaching in some countries, such as Chile, values of 0.5 kg of antibiotics for each kg of salmon produced [39]. Due to the extensive use of therapeutic antibiotics in aquaculture systems, reports on the monitoring of ECs, including antibiotic residues, ARGs, and ARB, have also increased, with a positive correlation between the use of antibiotics in aquaculture and the presence of these ECs [40,41]. Even more, the abundance of ARGs in aquaculture systems shows a positive correlation with the density and pond age in shrimp culture systems [41]. For almost twenty years, the effect of antibiotic residues, ARGs, and ARB has been of concern from a microbiological point of view since the presence of antibiotics in marine sediments can affect waste breakdown and influence the ecological structure of microbial communities [42]. An example of this was reported by Zheng and coworkers [43] when the presence of lincomycin, erythromycin, and sulfadiazine induced an inhibitory effect on perchlorate degradation by microorganisms from mangrove sediments. On the other hand, these ECs can favor the growth of a particular type or functional group of microorganisms, with the ability to grow in the presence of these contaminants, produced with this an imbalance in the aquatic microbiome [44].

Several approaches have been proposed to eliminate antibiotics in effluents from the treatment of domestic and industrial wastewater [45,46,47], including the use of constructed wetlands to remove micropollutants from aquaculture effluents with removal efficiencies of more than 87% [48]. The use of wetland systems with plant species (*Iris pseudacorus* and *Phragmites australis*) with high-rate removal capacity of emerging contaminants such as enrofloxacin, sulfamethoxazole, and ARGs has been proposed as an alternative to recover water from aquaculture effluents [49].

Mangroves, considered natural wetlands, are of great importance for coastal aquaculture systems, since this ecosystem supplies environmental services such as nursery grounds and shelter for fish, crabs, and shrimp, the maintenance of biodiversity, and water conservation [50]. These characteristics of mangroves have been used for years to facilitate the growth of economically important species in coastal aquaculture. Nevertheless, the conversion of mangrove areas into aquaculture farms produces a significant loss of the ecosystem [51]. The rapid development of aquaculture has been identified as “the blue revolution”, where the emission of blue carbon is produced by the deforestation of mangroves [52]. Nonetheless, mangroves are still considered an excellent option to filter effluents from coastal aquaculture systems and even remove ARGs from these effluents [24]. The impact on mangrove microbial communities due to antibiotics and ARGs from aquaculture is still under study. Recently, an association between chloramphenicol resistance genes in water and sediments in a mangrove area and the pressure in the presence of aquaculture ponds was reported [36]. Moreover, high concentrations of ciprofloxacin and norfloxacin (>250 ng·g^−1^) were reported in sediments of mangroves near mariculture farms, but a positive correlation between ARG abundance and antibiotic concentration in this environment was only found for ciprofloxacin [35]. From this, a relationship between this activity and the presence of ECs in mangrove ecosystems has been suggested.

#### 3.1.3. Livestock Breeding

The upsurge of antibiotic resistance due to the use of antibiotics in veterinary therapies has increased the cost of cattle production, along with a concern for the effect that the use of antibiotics can have on human health [53]. Since 90% of the antibiotics administrated to animals are excreted in the form of the original compound or metabolites via feces or urine, this is one of the forms in which the antibiotics are released to the environment [54,55,56]. Nevertheless, the impact of livestock breeding in the presence of antibiotics, ARGs, and ARB in several environments and their effect in these ecosystems still require more studies.

It has been reported that microbial communities found in wastewater from livestock breeding have a significant effect on modifications of the resistome (defined as the set of host genes with non-redundant function in resistance [57]) identified in waterbodies where these wastewaters are discharged [58]. The development of human activities, such as livestock breeding in the vicinity of estuarine environments, has been related to the increase in antibiotics of the quinolones group (in a range of concentrations of 0.276 to 5.229 ng·g^−1^) used in veterinary medicine [37]. Extensive animal breeding can be related to the dispersion of animals in large areas, with the consequent distribution of antibiotics and residues on a broader area than in intensive breeding systems. On the other hand, soil pollution with ARB and ARGs to antibiotics such as tetracycline and sulfonamide has been related to the use of manure from intensive chicken breeding systems to improve soil productivity [59]. Moreover, the risk that veterinary antibiotics present in soils reach groundwater by lixiviation is always imminent and has been reported to depend on the physicochemical characteristics of both antibiotics and soil particles [60]. On the other hand, the presence of antibiotic compounds and MDR bacteria in water samples from rivers close to livestock breeding activities has been found to be related to this activity [61,62]. Although the relationship between livestock breeding and the presence of ECs in mangroves has not been reported yet, the presence of antibiotic-related ECs in mangroves located close to rural communities is likely to be increased due to animal breeding, as has been described by other anthropogenic and economic activities’ development by the communities [63].

#### 3.1.4. Urbanization and Tourism

The effect of urbanization in the presence of ECs in mangroves is one of the less studied human activities. However, increased urbanization produces changes in land cover, biogeochemical processes, hydrological systems, biodiversity, and climate [64,65]. One of the clearest effects of rapid urbanization is its impact on the concentration of carbon dioxide (CO_2_) emissions in the short and long term [66], which is a factor related to global warming. This negative effect of urbanization is closely related to one of the most critical ecosystem services of mangroves, which counter the concentration of greenhouses gasses by CO_2_ sequestration from the atmosphere, known as blue carbon [2,3]. Nevertheless, urbanization brings high rates of CO_2_ emissions and a diversity of emerging contaminants such as antibiotics, which represent an accumulative risk for the health of the ecosystem [67].

Economic inversion in tourism has found a fertile area in countries with coastlines, especially with a warm climate and a large extension of sand beaches. The urbanization of these areas has allowed for an increase in the community’s income due to tourism [68]. The attraction of tourism is one of the recognized ecosystem services of mangroves [69]. Still, tourism is one of the main factors identified as responsible for the degradation of mangrove ecosystems [70]. Several reports describe the relationship between the presence of ECs in different environments and a high presence of touristic activities on those sites [71,72,73,74], and recently, this relationship has been reported in mangroves [21,38]. The enforcement of protection laws and environmental planning for mangrove protection from the negative effect of tourism, including the increase in ECs, must be priority actions by any country with mangrove ecosystems. However, these protection mechanisms might not be sufficient to stop the degradation process of mangroves [75].

### 3.2. Direct Effect of ECs in Mangrove Ecosystems—A Natural Biofilter for EC?

Although the presence of ECs in mangroves can be related to anthropogenic activities, there is no clear evidence that the contaminants present a negative impact on these ecosystems since it has also been reported that mangrove plants and their sediments reduce the presence of ARGs and ABs in sediments and water samples [23,24]. In Giaoqiao mangrove, a natural reserve of China that is under constant influence of aquaculture farms, sediments obtained far from the mangrove showed higher concentrations of antibiotics from two families (average values in µg·kg^−1^: 87.8—fluoroquinolones; 0.97—chloramphenicol) than sediments from mangrove areas (average values in µg·kg^−1^: 26.77—fluoroquinolones; 0.14—chloramphenicol) [23]. On the other hand, the abundance of six ARGs (*mtrA*, *rpoB*, *rpoC*, *rpsL*, *ef-Tu*, and *parY*) and the target alteration (as resistant mechanism) were significantly lower in mangrove sediment than in non-mangrove sediment [24]. The mechanisms used by mangrove plants to decrease the presence of these ECs in these environments remain a black box. Some works discarded the role of plants in this process, as was observed by Santos and co-workers [32], where the antibiotics added to constructed wetlands with *Phragmites australis* were removed but were not found in the plant’s roots. The latter supports the idea of a more active microbial degradation process for the ECs presented in these environments, as has been suggested before [76]. Another piece of evidence aligned with the idea was observed in constructed wetland biofilms, where the removal rate of ARGs related to antibiotics such as tetracycline, sulfonamide, macrolides, and penicillin reached more than 2 log values and was correlated with microorganisms related to ammonium and organic matter degradation [77].

Even so, when the ECs of interest are antibiotics, the role of plants has been described as vital for their removal [33]. The complex and competitive ecosystem present in mangrove sediments, where microorganisms are in constant interaction with mangrove plants, can explain the removal of ARB and ARG contaminants. In mangrove sediments, there is a continuous exchange of nutrients between microorganisms and plants, from simply oxygen released by the plants’ root to root exudates including sugars, amino acids, and other organic compounds, which stimulate the metabolism of rhizospheric microorganisms [78]. These plant–microbe interactions, along with the physicochemical properties of mangrove sediment, promote a synergic effect in plant and microbial metabolic pathways, where chemical complex compounds, such as antibiotics, can be converted to the simplest energy-generating compounds. Moreover, the correlation between mangrove sediment properties and the prevalence of ARGs in these environments was described by Zhao and co-workers [24].

Mangroves have been proposed as a solution for the decrease in EC concentration in coastal water bodies. The effects of these pollutants, including antibiotics presented in aquaculture effluents on mangrove plants, as well as the uptake mechanisms of plants to reduce these compounds, are not yet understood [79,80]. Additionally, in a metagenomic analysis of mangrove ecosystems with different degrees of pollution, a minor variation in the microbial communities between the ecosystems was observed, which indicates the high resilience capacity of this ecosystem against perturbations by pollutants [81]. On the other hand, the presence of antibiotic-resistant bacteria and genes in mangrove ecosystems has been reported in both pristine and polluted sites, and their presence has been related to the necessity of the bacteria to develop survival mechanisms for highly competitive conditions, such as those present in mangroves [82]. Nevertheless, the increase in selective pressure produced by the introduction of compounds outside this type of ecosystem continues to be under review, and it is presented as a new field of study. There are few studies related to the real impact of ECs such as antibiotics, ARB, and ARGs in the mangrove ecosystem.

## 4. Materials and Methods

According to PRISMA recommendations [83], we conducted a systematic review. Scopus, Web of Science, and Google Scholar databases were searched individually for the published papers. We focused on research related to mangrove ecosystems, without the restriction of the year and including only English language papers. The keywords, or strings, used were “mangrove antibiotic presence”, “mangrove antibiotic resistance”, “mangrove antibiotic resistance genes”, “mangrove antibiotic resistant bacteria”, “mangrove emerging contaminants”, “mangrove aquaculture”, “mangrove livestock”, “mangrove manure”, “mangrove wastewater”, “mangrove urbanization”, “mangrove service ecosystem”. The PRISMA guidelines were applied, and 77 articles were selected and included in this review.

## 5. Conclusions

The importance of mangrove ecosystems includes all the services that they provide to human communities. The relation between environmental degradation and anthropogenic activities has been studied for several years. The monitoring of emerging contaminants, including antibiotics, ARGs, and ARB in environments, has been proposed to determine the direct human impact in several environments. Nevertheless, monitoring these contaminants in mangroves has been studied for a few human activities, such as aquaculture, while other activities such as tourism, urbanism, and livestock breeding are still poorly understood. Moreover, the few EC analyses of AB, ARGs, and ARB in mangroves have been directed to propose these ecosystems as a solution to mitigate the presence of these pollutants, instead of analyses that correlate the presence of these ECs to human activities developed in the mangrove’s vicinity. Even more, the effect of these ECs on the physiology and health of this ecosystem remains unknown. The present review shows the lack of information on the actual effect of anthropogenic activities related to mangrove areas on physiology and functionality. The development of more studies in this area could warrant an accurate analysis of the human contribution to the degradation or conservation of these ecosystems.

## Data Availability

No original data are reported in this manuscript.
